# Evaluation of elderly women with uterin cervical cancer

**DOI:** 10.1002/cnr2.1570

**Published:** 2021-10-10

**Authors:** Özer Birge, Mehmet Sait Bakır, Ceyda Karadag, Selen Doğan, Hasan Aykut Tuncer, Tayup Simsek

**Affiliations:** ^1^ Department of Gynecology and Obstetrics Akdeniz University Antalya Turkey

**Keywords:** elderly women, evaluation, uterine cervical cancer

## Abstract

**Background:**

Uterine cervical cancer rates also increase with aging. Especially, the primary treatments of patients with cervical cancer include surgery, chemotherapy, and radiotherapy.

**Aim:**

Our aim is to discuss the effect of clinical and histopathological risk factors on survival in patients over 65 years old with invasive cervical cancer in the light of the literature.

**Methods and Results:**

The files of 60 patients aged 65 and over who were diagnosed, examined, and treated for invasive cervical uteri cancer between 2004 and 2021 by the gynecological oncology clinic of Akdeniz University were analyzed retrospectively after obtaining approval from the Akdeniz University ethics committee with the number KAEK‐110. Detailed written consent was obtained from all patients and their relatives for data analysis. Patients aged 65 and over who were diagnosed with invasive cervical uteri cancer at all stages who accepted treatment were included in the study. The patients who were not included in the study were those who did not accept treatment, did not continue their follow‐up regularly, were under 65 years of age, had preinvasive cervical lesion, had a second primary cancer, had an unknown stage, and died due to accidents or similar reasons.

When the demographic data of 60 cases were examined, the mean age was 70.5, the youngest age was 65, and the oldest age was 84. When we divided them into two groups by age groups, 76.7% were between 65 and 75 years old and 23.3% were over 75 years old. When the data of 60 patients who were referred to our hospital, which was a tertiary center in the 15 years duration, were examined, the mean disease‐progression free survival (PFS) of patients with locally advanced stage was 45 months, however, it was 4 months for metastatic patients, this difference was significant and a statistically significant difference was found between the two groups (*p*: .001). When the total survival was examined, the mean was 108.7 months in the locally advanced stage group, while it was 2.9 months in metastatic cases, and this difference was also statistically significant between the two groups (*p*: .001). When we divide the cases into two groups as between 65 and 75 and over 75 years of age, the mean age of disease‐free survival is 76.9 months in the 65–75 years old group, while 16 months in the 76–85 years old group, however, the *p* value of this difference in PFS between the two groups was not significant (*p*: 0.154). However, when the total survival was examined, it was seen that the mean was 140.4 in the 65–75 years old group, while it was 56 months in the 76–85 years old group and this difference was significant between the two groups (*p*: .046).

**Conclusion:**

In parallel with the increased population worldwide, advanced age cancer rates are increasing. In parallel with the population growth, it should be remembered that the patients over 65 years of age who were diagnosed with invasive uterine cervical cancer had difficulty in accessing screening tests, late diagnosis and inadequate treatment regimens due to concomitant diseases, resulting in recurrence in a short time and poor clinical symptoms due to short total survival.

## 1 BACKGROUND

The incidence of cervical uteri cancer varies in different regions of the world. Worldwide, it is the most common cancer among gynecological cancers; the third most common gynecological cancer after endometrium and ovarian cancer in the developed countries.^1^, ^2^ This fact reveals that cervical uteri cancer poses a more important problem in the developing countries. The situation in our country is similar to the developed countries. It is the third most common gynecological cancer. The lifetime risk is 1.1%, however, the incidence is reported as 4/100000 women.^1^

The most important risk factor is human papilloma virus (HPV) infection. HPV positivity was detected in 99% of invasive cancer cases.^3^ Other risk factors include smoking, immunosuppression, low‐socioeconomic level, and multipartner.^4^ In the patient infected with high‐risk HPV subtypes, the persistence and subsequent progression of the virus, especially the preinvasive lesions originating from the transformation region of the cervix uteri progress to invasive lesions over the years.^5^ The prolonged preinvasive period of the disease made it possible to screen the disease with a cervical swab. Today, countries that use cervical cancer screening programs effectively have reported a reduction in mortality due to cervical uteri cancer by more than 50%.^6^ Screening of cervical uteri cancer with HPV DNA, which is being used increasingly today has allowed screening with a higher sensitivity.

The reversal of the population pyramid in parallel with the population growth, especially in developed countries, and the rapid increase of the population aged 65 and over is an important problem. As getting older, the incidence of several types of cancer also increases. Cancer‐induced deaths at the age of 65 and above is the second reason of cardiovascular deaths. About 1 250 000 new cancer cases were detected in the USA in 2000 and it was reported that 80% of all cancers were observed at the age of 55 and above population. The 50% of tumors also occur in people over the age of 65, and this population is still 15% of the total population, but this rate is expected to be around 50% in 2021.^7^ The increased cancer incidence in the elderly may be explained in two important ways; molecular changes caused by aging and deficiency in the immune system increase the sensitivity of old tissues to carcinogens. And since carcinogenesis is a very long process, it is normal for cancer to occur in elderly population.

Caring for an elderly cancer patient is much more complicated than younger patients. Older patients often do not manifest specific symptoms and signs, making early diagnosis very difficult. Older people generally use more drugs than young people and this situation puts them at risk for drug–drug and drug‐disease interactions. Studies have shown that elderly patients are more hesitant than young people to make decisions about their own health. The old people want to rely on their families in such decisions, and this situation doubles the importance of the doctor's duty when confronting families who do not know how their decision will affect the patient.

Uterine cervical cancer rates also increase with aging. Especially, the primary treatments of patients with cervical cancer include surgery, chemotherapy, and radiotherapy. However, less aggressive treatment methods are preferred when compared with younger patients because of several concomitant diseases and body structure of elderly patients.^8^ We see that when cancers diagnosed in elderly patients, they are probably in advanced stage and in late admission.^9^, ^10^ Given the reasons for the advanced disease and late diagnosis of elderly cancer patients; it is reported that the screening tests are insufficient, the elderly care is inadequate, their access to the health system is more limited, and they cannot get effective health services.^11^

Our aim is to discuss the effect of clinical and histopathological risk factors on survival in patients over 65 years old with invasive cervical cancer, which is rare in literature.

## 2 MATERIAL AND METHODS

The files of 60 patients aged 65 and over who were diagnosed, examined, and treated for invasive cervical uteri cancer between 2004 and 2021 by the gynecological oncology clinic of Akdeniz University were analyzed retrospectively after obtaining approval from the Akdeniz University ethics committee with the number KAEK‐110. Detailed written consent was obtained from all patients and their relatives for data analysis. Patients aged 65 and over who were diagnosed with invasive cervical uteri cancer at all stages who accepted treatment were included in the study.

The patients who were not included in the study were the patients who did not accept treatment, did not continue their regular follow‐up visits, were under 65 years of age, had preinvasive cervical lesion, had a second primary cancer, had an unknown stage, and also patients who died in daily life due to traffic accidents, natural disasters or home accidents and primary causes other than cervical cancer were excluded from the study.

The staging was adjusted by FIGO criteria and the histological subtype classification by WHO guidelines (7.12).

Detailed information was given to all patients about the standard surgery of early stage cervical cancer, and then radical hysterectomy, bilateral salpingo‐oophorectomy, bilateral pelvic‐para‐aortic lymphadenectomy were performed in standard surgery for early stage cervical cancer, while adjuvant radiotherapy and/or chemotherapy combinations in the postoperative period. Primary radiotherapy and/or chemotherapy treatments were used sequentially or concurrent in locally advanced and metastatic cases. All patients were staged during the operation, tumor diagnosis, histology, regional lymph node evaluation, evaluation of biopsies from all areas, and cytology were performed by expert pathologists in a single pathology center for postoperative pathological evaluation. Adjuvant radiotherapy and/or platinum‐based chemotherapy was planned as sequential and/or concurrent treatment for all high‐risk patients after the operation. Patients with 4 cm or less stage IA‐IIA tumors were considered as early stage, while stage Ib3 and IIB‐IVA cases with masses greater than 4 cm were grouped as locally advanced stage and the patients with IVB as metastatic cervical uteri cancer.

It was observed that after the treatment, the patients continued their follow‐up visits every 3 months in the first 2 years and every 6 months in the next 3 years. Pelvic examination, transvaginal or transabdominal ultrasonography, serum tumor marker evaluation, and radiological evaluations were performed in all cases during follow‐up. Treatment regimens were applied carefully and best approach was presented for post‐operative follow‐up of elderly patients. Cases were excluded from the study if they did not continue follow‐up visits. The recurrences detected during the follow‐up were determined by imaging methods and pathological evaluations if required.

### 2.1 Statistical analysis

For the descriptive statistics, the mean, SD, median, min‐max values and frequencies were used, considering whether there was a normal distribution or not. The categorical data were expressed in numbers and percentages (%). Progression‐free survival (PFS) and overall survival (OS) were compared using the Kaplan Meier survival analysis. The log rank test was used for the effect of subgroups on survival. The statistical package for the Social Sciences 23 program was used in the analysis of the data. The *p* values in all tests were two‐sided, and when *p* values were lower than .05, it was considered to be statistically significant.

## 3 RESULTS

When the demographic data of 60 cases were examined, the mean age was 70.5, the youngest age was 65, and the oldest age was 84. When we divided them into two groups by age groups, 76.7% were between 65 and 75 years old and 23.3% were over 75 years old.

While the mean gravida was 4, the parity was 3, and the normal vaginal delivery rate was 87.5%. When the body mass index is calculated, the mean value was 24.33 (18.2–36.7), the smoking rate is on about 12 pack years, the average Ca‐125 value is 17.1: The smallest 2.8 and the highest 342. The mean hemoglobin value was 12.16 ± 1.42. Among 60 patients in the study, only 26.6% were literate and 78.4% had comorbid diseases, and the largest group was hypertension. When the patients were evaluated by cytological screening for cervical uteri cancer, it was seen that 38.3% did not undergone cytological screening tests in their lives, and only 3 (5%) of the 61.7% group who had screening tests had undergone HPV tests and the most common HPV was 16. When the histopathological types of invasive cancer are examined, it is seen that the most common group is the squamous cell group (75%, 45 cases), followed by the second common adenocarcinoma group (18.4%). Histopathological examination showed that lymphovascular stromal invasion was positive in 60% of the cases, 18.3% were negative and 21.7% of them did not provide information about lymphovascular stromal invasion in the pathology reports (Table 1).

**TABLE 1:** Descriptive demographic findings of the study group

		Number (*n*: 60)/%	
Age (median, range)		70.5	65–84
Age group	≥65–75	46	76.7%
	76–85	14	23.3%
Gravida (median, range)		4	2–9
Parity (median, range)		3	1–6
BMI (median, range)		24.22	18.2–36.7
Smoke (package year) (median, range)		12	0–32
Ca‐125 (median, range)		17.1	2.8–342
Hemoglobin value (mean, SD)		12.16	±1.42
Educational status	Yes No	16 44	26.6% 73.4%
Chronic illness	Not	13	21.6%
	Hypertension	15	25%
	Diabetic	11	18.3%
	Cardiac disease	5	8.3%
	Asthma	6	10%
	Thyroid	10	16.8%
HPV status	Yes	3	5%
	No	57	95%
HPV type	16	2	
	18	1	
	Others	2	
Cervical cytology status	Not	23	38.3%
	Normal	10	16.7%
	Ascus/Lgsil	7	11.7%
	Asc‐h/Hgsil	10	16.7%
	Agc	3	5%
	Cx ca	7	11.7%
Histology	SCC	45	75.0%
	Adeno	11	18.4%
	Others	4	6.6%
Lvsi status	Positive	36	60%
	Negative	11	18.3%
	Not	13	21.7%

Abbreviations: BMI; Body mass index; HPV, human papilloma virus; Lvsi: Lymphovascular stromal invasion; SCC; Squamous cell cancer.

When the clinical and pathological data were examined, it was observed that 49 of 60 cases (81.7%) were at locally advanced stage, three cases (5%) were metastatic, and eight cases (13.3%) were in the early stage. When we analyzed the stages one by one, we found that the group with stage IIb parametrial involvement constituted the largest group with 24 cases (40%). When the cases were evaluated by their treatment, in parallel with the stage, primary chemoradiotherapy was the most common group with 35 cases (58.3%) and two patients had in early stage so only surgery was sufficient and the other 20 cases (33.3%) who underwent surgery received adjuvant radiotherapy simultaneously or sequentially with platin based chemotherapy. Primary chemoradiotherapy was applied to 35 of 49 patients diagnosed with locally advanced cervical uteri cancer and 26 (74.3%) of them had recurrence and 9 (25.7%) patients had a response to complete treatment. First‐line chemotherapy was given to three cases (5%). Staging surgery was performed in three cases (5%) with early stage cervical uteri cancer as laparoscopic radical hysterectomy ± bilateral salpingoophorectomy ± bilateral pelvic and para‐aortic lymph node dissection operation. When all patients were evaluated in terms of the initiation time after the diagnosis for surgery, chemotherapy, or radiotherapy, it was seen that the mean time was 37.03 ± 20.7 days. When the lymphadenectomy materials of 22 cases who underwent surgical treatment were examined, it was found that 15 cases (68.1%) had negative lymph involvement, five cases (22.7%) had pelvic lymph node involvement, and two cases (9.2%) had pelvic and para‐aortic lymph nodes involvement. In 34 (56.7%) of the patients who were median followed up for about 60.9 months (0.57–193), recurrence was observed, and when we analyzed these recurrences, 22 (64.7%) were local and 12 (35.3%) were distant metastasis. It is observed that 30 (50%) cases died in a total of 193 months of follow‐up and the remaining 50% are still being followed up (Table 2).

**TABLE 2:** Descriptive clinical and pathological risk factors of the study group

Stage grouped	Early stage	8	13.3%
	Locally advanced	49	81.7%
	Metastatic	3	5%
Stage	Ib1	2	3.3%
	Ib2	6	10%
	Ib3	5	8.3%
	IIa1	2	3.3%
	IIa2	3	5%
	IIb	24	40%
	IIIa	1	1.7%
	IIIb	5	8.3%
	IIIc1	4	6.6%
	IIIc2	2	3.3%
	IVa	3	5%
	IVb	3	5%
Primary type of treatment	Surgery	2	3.3%
	Surgery + adjuvant RT	6	10%
	Surgery + adjuvant CRT	14	23.3%
	Pr. CRT	35	58.3%
	Pr. CT	3	5%
Laparoscopy status	Yes	3	5%
	No	57	95%
Lymphadenectomy status	Yes	22	36.6%
	No	38	63.4%
Lymph involvement	Negative	15	68.1%
	Pelvic positive	5	22.7%
	Pelvic and Para‐aortic positive	2	9.2%
Recurrence	Yes	34	56.7%
	No	26	43.3%
Recurrence locations	Locally	22	64.7%
	Distances	12	35.3%
Survival status	Death	30	50%
	Alive	30	50%
Time to start treatment (mean ± SD) (days)		37.03	±20.7
Follow‐up time (median) (months)		60.9	0.57–193

Abbreviations: CRT, chemoradiotherapy; CT, chemotherapy; RT, radiotherapy.

**FIGURE 1:**
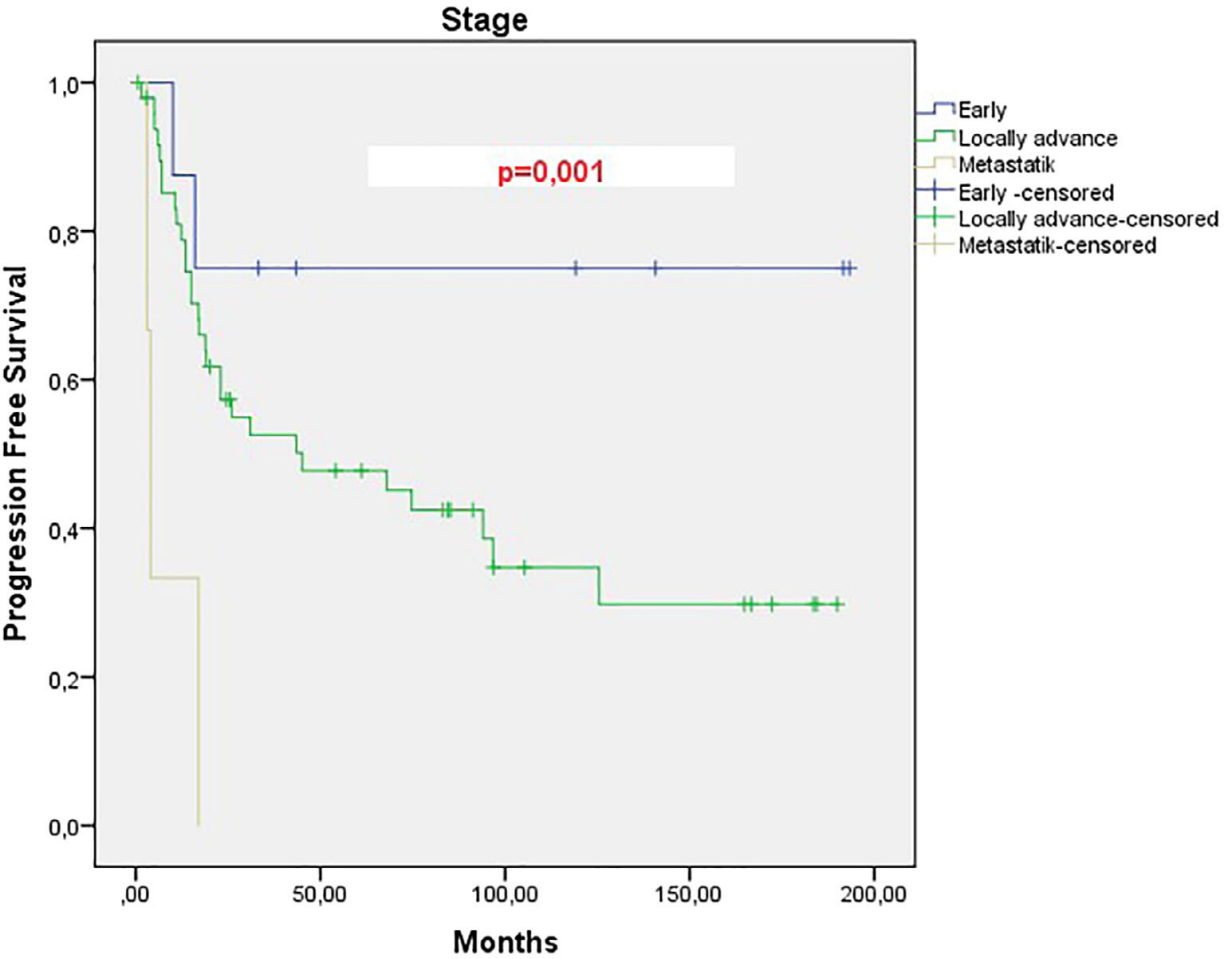
Progression‐free survival analysis for patients with cervical cancer according to the stage

**FIGURE 2:**
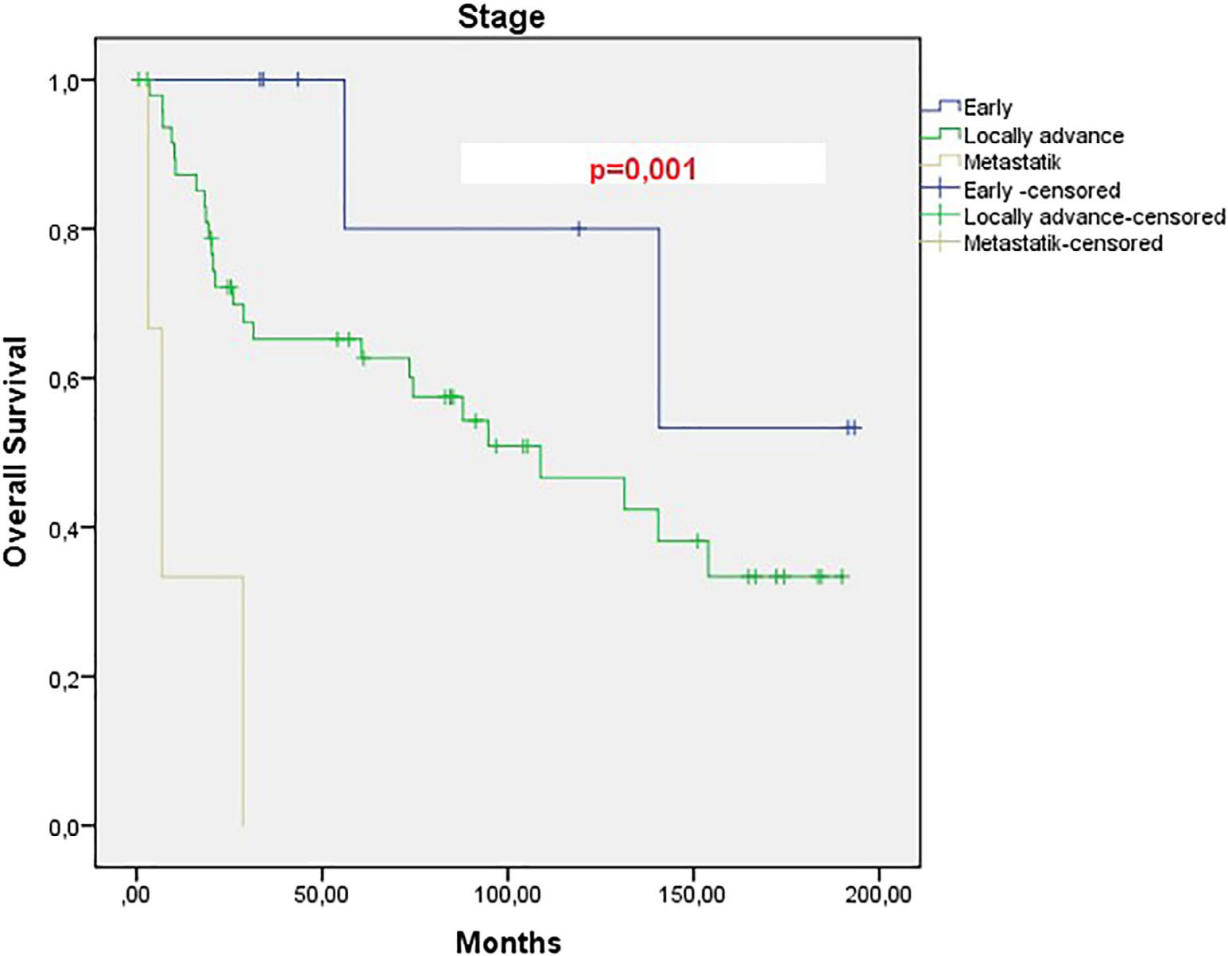
Overall survival analysis of patients with cervical cancer according to the stage

**FIGURE 3:**
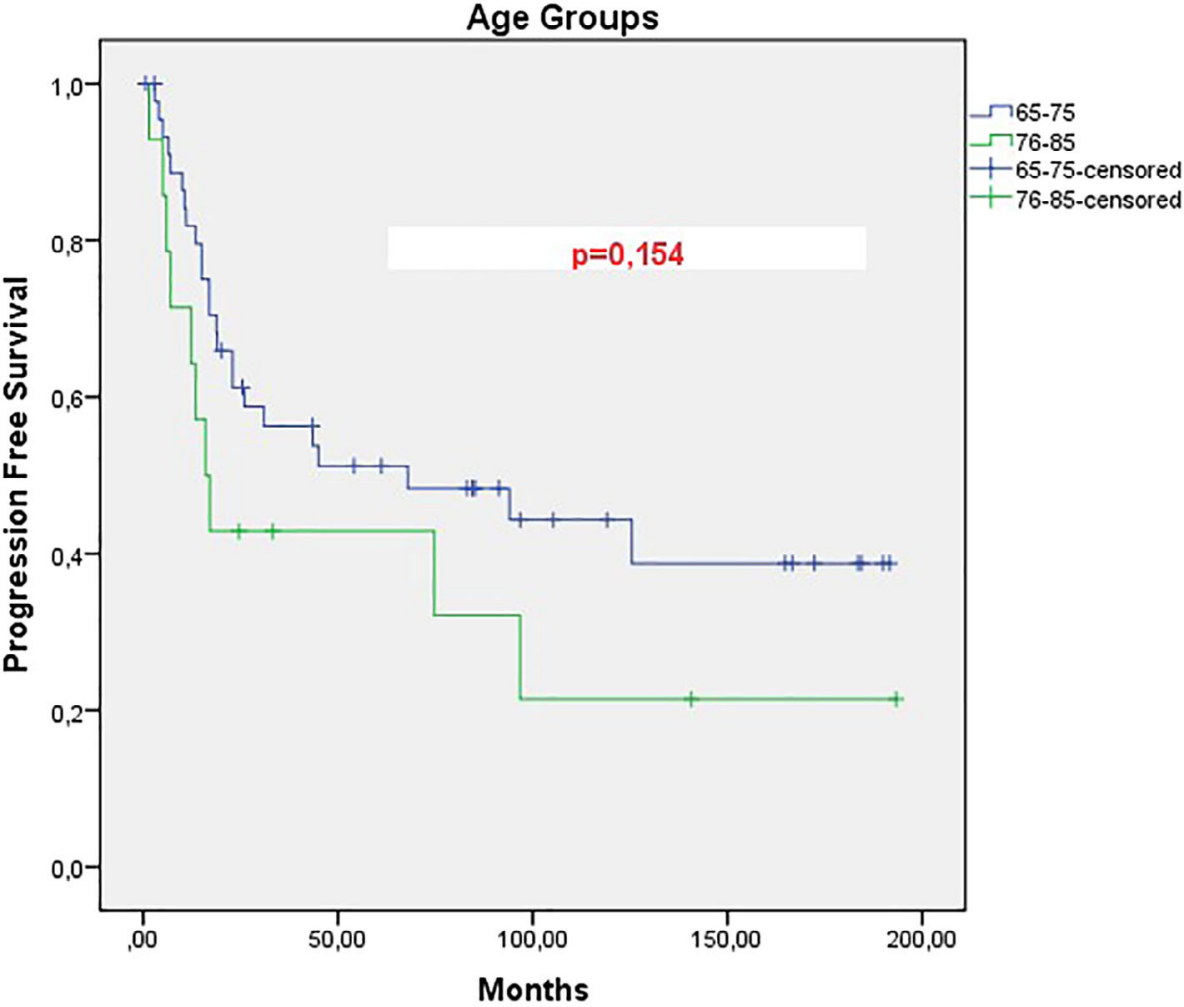
Progression free survival analysis according to age subgroups of our patients with cervical cancer

**FIGURE 4:**
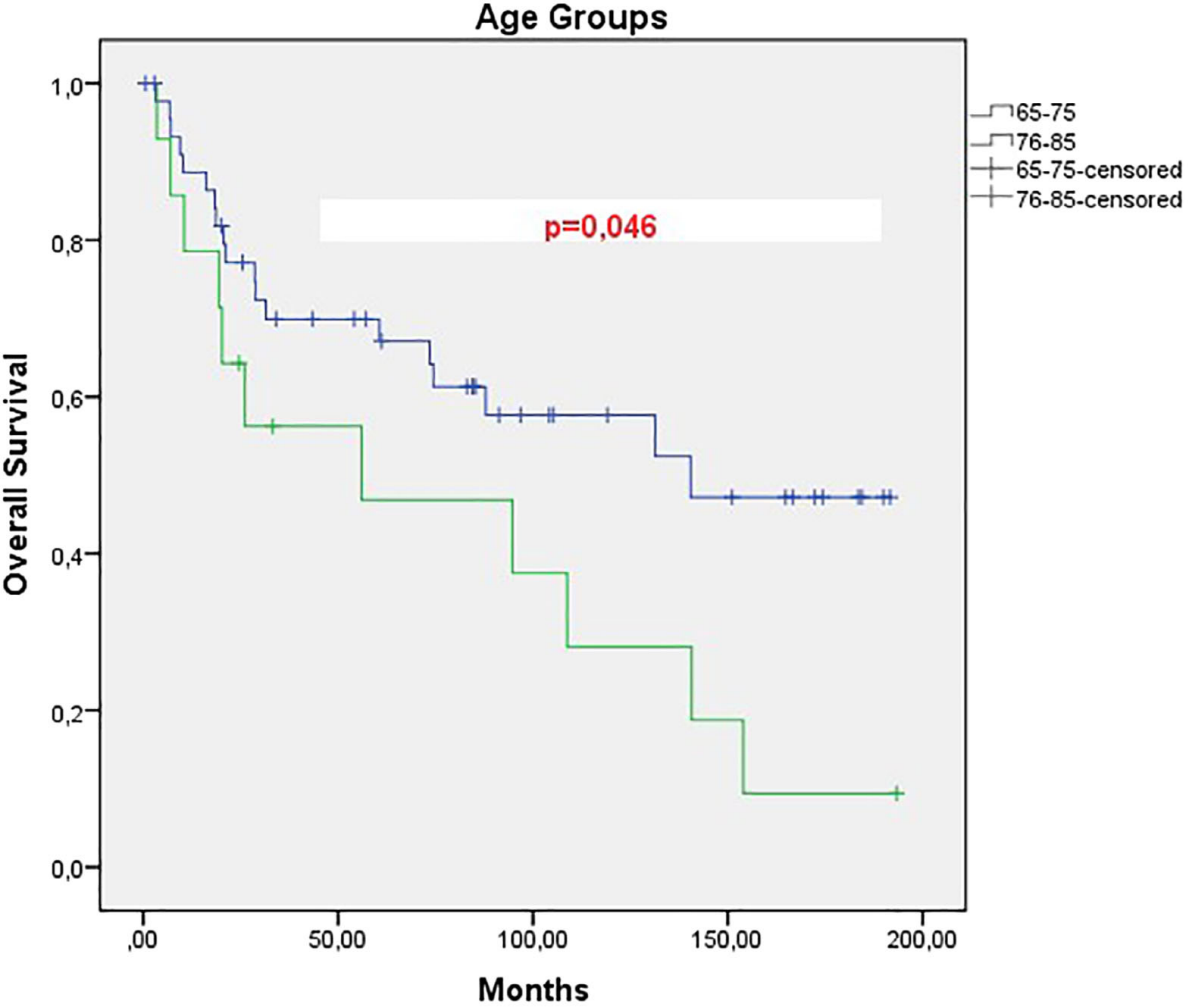
Overall survival analysis of our patients with cervical cancer according to age subgroups

When the data of 60 patients who were referred to our hospital, which was a tertiary center in the 15 years duration, were examined, the mean PFS of patients with locally advanced stage was 45 months, however it was 4 months for metastatic patients, this difference was significant and a statistically significant difference was found between the two groups. (*p*: .001).(Figure 1) When the total survival (OS) was examined, the mean was 108.7 months in the locally advanced stage group, while it was 2.9 months in metastatic cases, and this difference was also statistically significant between the two groups (*p*: .001).(Figure 2) When we divide the cases into two groups as between 65 and 75 and over 75 years of age, the mean age of PFS is 76.9 months in the 65–75 years old group, while 16 months in the 76–85 years old group, however, the *p* value of this difference in PFS between the two groups was not significant (*p*: 0.154).(Figure 3) However, when the total survival was examined, it was seen that the mean was 140.4 in the 65–75 years old group, while it was 56 months in the 76–85 years old group and this difference was significant between the two groups (*p*: .046).(Figure 4).

## 4 DISCUSSION

In this study, we studied the demographic, clinical, and pathological characteristics, disease‐free survival and OS and the effective risk factors in patients diagnosed with cervical uteri invasive cancer at the age of 65 years and older. Our study found that the 75–85 age group had a statistically significant difference in OS as compared to the 65–75 age group, but there was no statistically significant difference in progressive free survival, however there a difference was found in survival. The evaluation of the patients by their stages showed that a significant difference was found both in pfs and total survival, respectively, *p* values *p*: .001 and *p*: .001. Besides, we found that the mean age was 70.5 and 23.3% of them were over 75 years old. It was observed that only 13.3% of the patients in the study were in the early stage, especially 86.7% admitted during the late stage, and only 61.7% of all patients had a cervical cytology‐screening test in their life.

Cervical uteri cancer screening was introduced with PAP smear in the 1940s and this screening was considered as the most effective method among screening programs for all cancer types.^12^ Then, in the 1990s, liquid‐based cytology was introduced in practice as a screening method. Finally, after the relationship between HPV and cervical uteri cancer was understood clearly, combined screening tests including HPV DNA and cytology have been used since early 2000s.^8^, ^13^ The rate of HPV test positivity was 3.5% in the HPV‐based screening tests among 1 million 30–35 years old women. However, like worldwide, patients over 65 years old admit at more advanced stages because screening tests after 65 years old were discontinued.^13^ We see that the rate of patients who underwent HPV testing was 5% in our study, at very low levels. However, since the HPV screening test is not used routinely over the age of 65, it is difficult to say anything about the true positivity rate.

Cervical uteri cancer is the second most common gynecological cancer among women worldwide.^14^ Studies have shown that the mean age at diagnosis is 48 years, and it peaks after 40 years old. Worldwide, cervical cancer incidence is increased with aging, and even though it is detected at an early stage by screening tests, the incidence continues to rise. Each year, elderly patients account for more than 40% of deaths due to cervical uteri cancer.^15^ Because of the recent developments in medical and surgical methods in health sector, the increased life expectancy worldwide increases the incidence of some cancers in elderly population. ıt is estimated that the population above 65 years old increases by 23% every year.^9^ So, the elderly population increases and their concomitant comorbid diseases increase, the incidence of diagnosed cervical cancer cases also increase and their treatment cause additional diseases, their performance status, disease stages, and the effect of the treatment on relapse and survival should be meticulously considered.

The number of hospital admissions is decreasing, especially since the decrease in the frequency of sexual intercourse in advanced ages causes the most common symptoms of postcoital bleeding, discharge, and spotting to be hidden. Concealing the clinical symptoms of cervical uteri cancer in these elderly patients causes inadequate screening, diagnosis, and treatment. As a result of all these, since these patients apply to clinics at more advanced stages, it is stated that their clinical prognosis is worse, especially over 65 years of age.^16^, ^17^, ^18^

It has been stated that regular cervical cytology screening in the population over 65 years old reduces the invasive cancer incidence by 75% and also significantly reduces the mortality rates.^19^ Therefore, routine screening tests even when there is no typical clinical symptoms and signs contributes to early diagnosis.^20^ We know that the genital organs shrink physiologically with age. Of course, the cervix uteri volume decreases due to the physiological shrinkage of the anatomical stromal connective tissue structures of the normal cervix uteri. When we analyzed the clinical and pathological data of our patients diagnosed with cervical uteri cancer at an advanced age; as our clinical interpretation, we can say that local invasion of malignant invasive tumors developing in the cervix uteri is easier in a short time from these tissues with atrophic and decreased blood flow perfusion.

Several studies reported that cervical uteri cancer is detected in elderly population and diagnosed in advanced stages because of the delayed screening tests and its prognosis is poor.^21^, ^22^ Again, the studies conducted in developed societies, France and Japan showed that cervical uteri cancers in elderly women developed in the later stages because of the lack of screening tests, and in conclusion, causing poor survival and higher recurrence rates.^23^, ^24^ Another study conducted in the developed society, Canada, reported that cervical cytological screening should not be discontinued in elderly population because invasive cancer cases in older ages are at more advanced stages and their prognosis is poor.^25^ In our developing society, screening is discontinued in 65‐year‐old patients with twice‐negative screening tests, like applied in most countries of the world. In line with the literature, our study in this population showed that the cervical cytological screening rates are 61.7%, and HPV scanning, which is the important risk factor, is only 5%, and 86.7% of our patients are diagnosed at advanced stages due to the deficiencies in this screening strategy. Again in line with our study results, a large population‐based SEER data study on the women diagnosed with 59 848 invasive cervical cancer between 1998 and 2002 evaluated by stage showed that 50% of them were locally advanced, 30% early stage, 9% metastatic stage, and 9% could not be identified.^26^ Considering all these results, there is a relationship between the last smear scan and the length of the period to diagnosis and the advanced stage, and this relationship is significantly statistically different. Therefore, it is stated that routine screening tests in older women will contribute to the earlier diagnosis.^26^

Especially in underdeveloped and developing countries, they are most frequently diagnosed in the locally advanced, then early stage and least in the metastatic stage.^1^ In the early stage, surgical treatment is the main treatment option, followed by adjuvant radiotherapy based on risk factors, mostly as brachytherapy ± chemotherapy. Brachytherapy is the main component of treatment in risky patients rather than wide area radiotherapy in early stage and locally advanced invasive lesions. Primary chemoradiotherapy is the main treatment modality for cancers that are commonly detected in locally advanced stages.^2^ In line with the literature, our study showed that most of them were in advanced stages and 78.4% had comorbid diseases, so, primary chemoradiotherapy as conservative method was applied most commonly (58.3%) for clinical symptoms and complaints at therapeutic doses. The second most common was postoperative adjuvant chemoradiotherapy.

When the distribution of 60 patients over 65 years old was evaluated by histology, squamous cell cancer was the most common (75%), while adenocarcinoma was the second most common histological types (18.4%). In line with our study, literature showed that the most common histological subtype, was squamous cell carcinoma (69%), followed by adenocarcinoma (25%).^27^

The current FIGO 2018 staging system is in parallel with the survival times of the patients.^28^ The results of the American Cancer Society showed that the five‐year survival rate in cervical uteri cancer was 92% in cases with localized disease, 56% in locally advanced cases, and 17% in metastatic cases (30–1669).^29^ Our study showed that lymph node involvement is an important criterion in prognosis, and its rate was 31.9%, and in 9.2% of them had pelvic and para‐aortic lymph node involvement positivity at the same time. It was observed that recurrence was identified in 56.7% of the cases after the treatment and local regional recurrence was the most common group (64.7%). When we analyzed the disease‐free survival rates, no significant difference was found between the two groups, 65–75 years old group, and over 75 years old group. However, when we separated them by stages, a significant difference was found between patients with locally advanced stage and metastatic patients (*p*: .001). When evaluated in terms of survival, it was found that there was a significant difference between locally advanced and metastatic groups (*p*: .001), and a statistically significant difference was found between the two groups 65–75 years old and over 75 years old in terms of survival (*p*: .046). It was observed that 50% of the cases died during an average of 60.9 (0.57–193) months follow‐up.

Age constitutes the most important group in determining the treatment of invasive cervical cancer, however, there are publications stating that the advanced stage, treatment modality and the time of treatment initiation are important.^30^, ^31^, ^32^ One of these studies which did not give an average time to start treatment reported that early treatment initiation is required and the rate of delayed treatment was high with a rate of 9.91%, especially in patients over 75 years of age, and delayed treatment caused recurrences in a short time and decreased total survival rates.^33^ In our study, the mean time to start treatment was estimated as 37.03 ± 20.7 days, and we might say it is late even though it is not a standard time to start treatment.

Unfortunately, the pros and cons of cervical uteri cancer screening in elderly patients has not been clarified in the literature and scientific guidelines. Today, three consecutive negative cytology tests or two consecutive HPV‐based cotest results in 10 years, including the last test within 5 years, are considered sufficient to discontinue screening for 65 years of age. Because, it is considered that these patients who have reached the age limit of 65 are not under increased risk when the benefit‐harms and risk assessment is performed. Of course, the studies stated that high‐grade cervical lesions are rare in women who have had screening tests, but women who have never undergone screening have high incidence and much higher mortality rates due to high‐grade lesions and invasive cancer, demonstrating the importance of screening these women for cervical lesions.^34^, ^35^ We accepted the age of 65, the age at which the screening is discontinued, as the limit and randomized our patients into two groups, and we saw that the incidence of invasive cancer increased by aging and, unfortunately, if the stage is advanced, we might say that the screening should be considered in patients having clinical symptoms and findings.

The limitations of our study are that it was a retrospective study, the number of cases (60 cases) was small, and there is no control group consisted of 65 years old and younger subjects who continue screening, therefore we could not demonstrate the effects of screening and treatment strategies clearly.

In conclusion, in parallel with the increased population worldwide, advanced age cancer rates are increasing. In parallel with the population growth, it should be remembered that the patients over 65 years of age who were diagnosed with invasive uterine cervical cancer had difficulty in accessing screening tests, late diagnosis and inadequate treatment regimens due to concomitant diseases, resulting in recurrence in a short time and poor clinical symptoms due to short total survival. Especially the patients, their families and clinicians should be careful about cervical uteri cancer and new individualized preventive screening and treatment strategies should be developed.

## CONFLICT OF INTEREST

The authors declare no potential conflict of interest.

## AUTHOR CONTRIBUTIONS

**Ö.B.;** Conceptualization (equal); data curation (equal); formal analysis (equal); funding acquisition (equal); investigation (equal); methodology (equal); project administration (equal); resources (equal); software (equal); supervision (equal); validation (equal); visualization (equal); writing – original draft (equal); writing – review and editing (equal). **M.S.B.;** Conceptualization (equal); resources (equal); software (equal); validation (equal); visualization (equal); writing – original draft (equal); writing – review and editing (equal). **C.K.;** Conceptualization (equal); formal analysis (equal); funding acquisition (equal); investigation (equal); methodology (equal); project administration (equal). **S.D.;** Data curation (equal); formal analysis (equal); software (equal); supervision (equal); validation (equal); visualization (equal). **H.A.T.;** Conceptualization (equal); data curation (equal); supervision (equal); validation (equal); visualization (equal). **T.Ş.;** Resources (equal); validation (equal); writing – original draft (equal); writing – review and editing (equal).

## ETHICS STATEMENT

The study was conducted in accordance with the Declaration of Helsinki (as revised in 2013). The study was approved by the ethics committee of the University of Akdeniz University Hospital # 2020 KAEK‐110 and individual consent for this retrospective analysis was waived.

## Data Availability

The data that support the findings of this study are available from the corresponding author upon reasonable request.
